# Association of Sleepwalking and REM Sleep Behavior Disorder With Parkinson Disease in Men

**DOI:** 10.1001/jamanetworkopen.2021.5713

**Published:** 2021-04-13

**Authors:** Xinyuan Zhang, Samantha A. Molsberry, Milena Pavlova, Michael A. Schwarzschild, Alberto Ascherio, Xiang Gao

**Affiliations:** 1 Department of Nutritional Sciences, The Pennsylvania State University, University Park; 2 Department of Nutrition, Harvard T.H. Chan School of Public Health, Boston, Massachusetts; 3 Department of Neurology, Brigham and Women’s Hospital, Boston, Massachusetts; 4 Department of Neurology, Massachusetts General Hospital, Boston; 5 Department of Epidemiology, Harvard T.H. Chan School of Public Health, Boston, Massachusetts; 6 Channing Division of Network Medicine, Brigham and Women’s Hospital, Harvard Medical School, Boston, Massachusetts

## Abstract

**Importance:**

Previous studies conducted among patients with Parkinson disease (PD) reported that parasomnias other than rapid eye movement (REM) sleep behavior disorder (RBD), particularly sleepwalking (SW), are associated with PD severity. However, it remains unclear whether the presence of SW is associated with altered odds of having PD in a population-based study.

**Objective:**

To evaluate whether probable SW, either alone or co-occurring with probable RBD, is associated with higher odds of PD in men.

**Design, Setting, and Participants:**

This cross-sectional study included 25 694 men from the Health Professionals Follow-up Study, a population-based cohort of male health professionals in the US with information on probable SW and probable RBD. Data collection took place between January 2012 and June 2018, and data analysis took place from July 2020 to October 2020.

**Exposures:**

Probable SW and probable RBD were measured by questions adapted from the Mayo Sleep Questionnaire in 2012.

**Main Outcomes and Measures:**

PD, confirmed after review of medical records by a movement disorder specialist.

**Results:**

Of the 25 694 studied men (mean [SD] age, 75.6 [7.4] years), 223 (0.9%) had probable SW, 2720 (10.6%) had probable RBD, and 257 (1.0%) had PD. After adjusting for potential confounders (eg, age, smoking, caffeine intake, chronic disease status, and other sleep disorders), compared with individuals without probable SW and probable RBD, participants with probable SW, probable RBD, and both probable SW and probable RBD had higher odds of PD, (probable SW: odds ratio [OR], 4.80; 95% CI, 1.61-14.26; probable RBD: OR, 6.36; 95% CI, 4.83-8.37; both probable SW and probable RBD: OR, 8.44; 95% CI, 3.90-18.27).

**Conclusions and Relevance:**

In this cross-sectional study of a male population, probable sleep parasomnias, including both SW and RBD, were associated with higher odds of having PD. PD-related neurodegeneration may impair arousal regulation during sleep.

## Key Points

**Question:**

Is the presence of sleepwalking, either alone or co-occurring with rapid eye movement (REM) sleep behavior disorder, associated with higher odds of having Parkinson disease in men?

**Findings:**

In this cross-sectional study of 25 694 men from the Health Professional Follow-up Study, both probable sleepwalking and REM sleep behavior disorder were significantly associated with higher odds of having Parkinson disease.

**Meaning:**

This study suggests that sleep regulation might be associated with Parkinson disease–related neurodegeneration.

## Teaser

This cross-sectional study evaluates whether probable sleepwalking, either alone or co-occurring with probable rapid eye movement sleep behavior disorder, is associated with higher odds of Parkinson disease in men.

## Introduction

Parkinson disease (PD) is the fastest-growing neurological disease in prevalence, disability, and deaths globally since 1990.^1^ PD is a movement disorder, but several nonmotor features have been frequently observed among patients with PD, which may be associated with progressive neurodegeneration.^2^ Identifying nonmotor symptoms is critical for understanding disease pathology and clinical significance.^3^

Rapid eye movement (REM) sleep behavior disorder (RBD) is one of the most extensively studied nonmotor features of PD.^2,3^ Small case series and descriptive studies conducted among patients with PD have suggested that sleepwalking (SW), a parasomnia that emanates from non-REM sleep (usually slow-wave sleep), appeared to have a high prevalence in patients with PD (approximately 10%).^4,5^ Given that SW is generally considered a rare condition in adults (1.0%-2.3%, according to a meta-analysis^6^), this prevalence in patients with PD appears unusually high, but to our knowledge, no study to date has included a group of individuals without PD in direct comparison for risk estimation. Furthermore, in additional studies of patients with PD, the presence of non-REM parasomnias was also associated with worse symptoms, cognitive impairment, and depression,^7,8^ which could be associated with dysfunction of arousal and motor control.^4,8^ Therefore, we conducted a cross-sectional study using data from the Health Professionals Follow-Up Study (HPFS) to examine the association between probable SW and probable RBD, measured by a validated questionnaire, and PD in more than 25 000 US men.

## Methods

We followed the Strengthening the Reporting of Observational Studies in Epidemiology (STROBE) reporting guideline for cross-sectional studies. This study was based on the HPFS, a cohort that started in 1986 with 51 529 male health professionals in the United States. The study protocol was approved by the institutional review boards (IRBs) of the Brigham and Women’s Hospital and the Harvard T.H. Chan School of Public Health, and the IRBs allowed participants’ completion of questionnaires to be considered implied consent. Participants received mailed questionnaires every 2 years; in 2012, it included questions on probable SW and probable RBD for the first time. Among the 27 093 participants who responded to the probable SW and probable RBD questions, 1343 individuals (5.0%) were excluded because they did not have a sleep partner to answer questions concerning the participant’s probable SW and probable RBD (eFigure in the Supplement).

Detailed procedures for PD ascertainment have been described previously.^9^ Briefly, PD cases that occurred before 2012 were confirmed by a movement disorder specialist reviewing medical records for those who reported having received a PD diagnosis on the regular follow-up questionnaires.^9^

The adapted Mayo Sleep Questionnaire was used to assess probable SW and probable RBD by asking whether the participant’s sleep partner had noticed the participant walking or acting out dreams while sleeping.^2,10^ In a validation study (n = 176), the question of probable SW had 100% sensitivity and 86% specificity, while the question of probable RBD had 98% sensitivity and 74% specificity relative to polysomnography results.^10^ Based on answers to these 2 questions, participants were categorized in 4 groups: no probable SW or probable RBD, probable SW only, probable RBD only, and both probable SW and probable RBD.

Potential confounders were assessed via questionnaires, including age, smoking status, weight and height, diet (eg, alcohol and caffeine consumption), history of major chronic diseases (eg, hypertension and diabetes), restless legs syndrome (RLS), and sleep-related parameters (eg, sleep duration, insomnia symptoms, and use of hypnotics).^11,12^

### Statistical Analysis

Statistical analyses were performed from July to October 2020, using SAS version 9.4 (SAS Institute). Statistical analyses were 2-tailed (α < .05). Odds ratios (ORs) with 95% CIs from the Firth penalized logistic regressions were estimated, and *P* values were calculated using the Wald test, adjusting for age, smoking status (never, past, or current), body mass index (calculated as weight in kilograms divided by height in meters squared; <21, 21-24.9, 25-29.9, 30-34.9, or ≥35), alcohol consumption (0, 1-9.9, 10-19.9, 20-29.9, or ≥30 g/d), caffeine consumption (mg/d), hypertension (yes or no), diabetes (yes or no), total sleep duration (<6 hours, 6-8 hours, >8 hours), excessive daytime sleepiness (rarely or never, some days, or most days), hypnotics use (yes or no), antidepressant use (yes or no), and RLS (yes or no). When the case number is small, a penalized logistic regression allows the computation of reliable, finite estimates of coefficients if separation in a data set is observed,^13^ and model fitness was evaluated by bootstrap validation adjusting for optimism to exclude the risk of overfitting. The model fitness ranged from good to very good; optimism-corrected C-statistic ranged from 0.739 to 0.851 (eTable 1 in the Supplement). We used missing values indicators for individuals missing information on a covariate so that each model included the same number of individuals. We were aware that this method could be subject to residual confounding, but we determined that this was likely to be modest because of the small number of individuals missing information on each covariate (eTable 2 in the Supplement).

Interaction between probable SW and probable RBD was examined by multiplying the 2 factors and entering them in the model after adjusting for the aforementioned covariates. We further tested an interaction between these sleep disorders and age, smoking, and caffeine consumption in relation to the odds of having PD, and we examined the association of disease duration by conducting a subgroup analysis stratified by years since PD diagnosis. Because RLS, another common sleep and movement disorder, was associated with a risk of PD in the HPFS,^11^ we also performed a sensitivity analysis by excluding participants with RLS.

## Results

At the time of assessment, the 25 694 participants had a mean (SD) age of 75.6 (7.4) years. A total of 223 (0.9%) had probable SW, 2720 (10.6%) had probable RBD, and 257 (1.0%) had documented cases of PD. Participants with probable SW or probable RBD were more likely to have longer sleep duration compared with those without sleep parasomnias (18 [17.1%] or 462 [17.8%] vs 2407 [12.7%]), prevalent daytime sleepiness (20 [19.1%] or 466 [18.0%] vs 2291 [12.1%]), hypnotics use (42 [39.3%] or 497 [19.3%] vs 3224 [17.0%]), and RLS (9 [9.7%] or 134 [6.3%] vs 961 [5.7%]) (Table 1). The excluded population were older and had more sleep complaints (eg, excessive daytime sleepiness, hypnotics use) compared with the included population (eTable 3 in the Supplement). In our cohort, 8 of 11 (72.8%) of patients with PD and probable SW also had probable RBD.

**Table 1.** Baseline Characteristics of Men in the Health Professionals Follow-up Study, 2012

Characteristic	Participants, No. (%), by probable sleep behavior disorders			
	Neither (n = 22 866)	Probable SW (n = 108)	Probable RBD (n = 2605)	Probable SW and probable RBD (n = 115)
Age, mean (SD), y	75.6 (7.4)	76 (7.4)	75.3 (7.1)	75.1 (7.4)
BMI, mean (SD)^a^	24.6 (7.1)	24.2 (7.1)	25.1 (6.2)	23.6 (7.6)
Smoking status				
Never	11 315 (52.5)	46 (46.5)	1314 (52.4)	56 (50.0)
Past	9672 (44.9)	49 (49.5)	1118 (44.6)	50 (44.6)
Current	578 (2.7)	4 (4.0)	77 (3.1)	6 (5.4)
Alcohol consumption, mean (SD), g/d^a^	13 (15.8)	14.6 (20.3)	13.4 (16)	14.9 (18.3)
Caffeine consumption, mean (SD), mg/d^a^	147.9 (137.6)	141.3 (142.2)	138.2 (130.8)	167.1 (165.8)
Hypertension	11 277 (49.3)	55 (50.9)	1293 (49.6)	60 (52.2)
Diabetes	2659 (11.6)	12 (11.1)	290 (11.1)	19 (16.5)
Total sleep duration, h				
<6	550 (2.9)	6 (5.7)	68 (2.6)	7 (6.1)
6-8	15 996 (84.4)	81 (77.1)	2060 (79.5)	74 (64.4)
>8	2407 (12.7)	18 (17.1)	462 (17.8)	34 (29.6)
Excessive daytime sleepiness	2291 (12.1)	20 (19.1)	466 (18.0)	31 (27.4)
Hypnotics use	3224 (17.0)	42 (39.3)	497 (19.3)	39 (33.9)
Antidepressant use	392 (1.7)	2 (1.9)	89 (3.4)	7 (6.1)
Restless leg syndrome	961 (5.7)	9 (9.7)	134 (6.3)	14 (16.5)

Abbreviations: BMI, body mass index (calculated as weight in kilograms divided by height in meters squared); RBD, rapid eye movement sleep behavior disorder; SW, sleepwalking.

^a^ Value is age-adjusted.

Participants with single or both probable parasomnias were compared to address misclassification. Individually, both probable SW and probable RBD were significantly associated with PD in the fully adjusted model (probable SW: adjusted OR, 4.80; 95% CI, 1.61-14.26; probable RBD: adjusted OR, 6.36; 95% CI, 4.83-8.37) (Table 2). Participants with both probable SW and probable RBD had an OR of 8.44 (95% CI, 3.90-18.27), although the multiplicative interaction between probable SW and probable RBD was not significant. Similar results were observed after excluding participants with RLS (Table 2). No significant interaction was found with age, smoking status, or caffeine consumption.

**Table 2.** Joint Analysis of Probable Sleep Behavior Disorders in Association With Parkinson Disease^a^

Model	Probable sleep behavior disorders, OR (95% CI)^a^			
	Neither (n = 22 866)	Probable SW (n = 108)	Probable RBD (n = 2605)	Probable SW and probable RBD (n = 115)
Case, No.	145	3	101	8
Model 1^b^	1 [Reference]	5.20 (1.77-15.32)	6.51 (5.04-8.42)	12.79 (6.21-26.34)
Model 2^c^	1 [Reference]	5.37 (1.83-15.79)	6.90 (5.33-8.93)	12.55 (6.05-26.04)
Model 3^d,e^	1 [Reference]	4.80 (1.61-14.26)	6.36 (4.83-8.37)	8.44 (3.90-18.27)
Excluding 1118 participants with RLS	1 [Reference]	5.79 (1.95-17.19)	6.60 (4.95-8.79)	10.20 (4.47-23.23)

Abbreviations: OR, odds ratio; RBD, rapid eye movement sleep behavior disorder; RLS, restless legs syndrome; SW, sleepwalking.

^a^ Missing values indicators for individuals missing information on a covariate were used.

^b^ Adjusted for age and smoking status (never, past, or current smoker).

^c^ Further adjusted for body mass index calculated as weight in kilograms divided by height in meters squared (<21, 21-24.9, 25-29.9, 30-34.9, or ≥35), alcohol consumption (0, 1-9.9, 10-19.9, 20-29.9, or ≥30 g/d), caffeine consumption (mg/d), hypertension (yes or no), and diabetes (yes or no).

^d^ Further adjusted for total sleep duration (<6 hours, 6-8 hours, >8 hours), excessive daytime sleepiness (rarely or ever, some days, or most days), hypnotics use (yes or no), antidepressant use (yes or no), and restless leg syndrome (yes or no).

^e^ *P* values for interaction between probable SW and RBD = .06.

After stratification by PD duration, probable SW without probable RBD was only significantly associated with PD among those with disease duration of 4.1 years to 8 years (OR, 6.27; 95% CI, 1.32-29.81) and more than 8 years (OR, 12.05; 95% CI, 3.22-45.04), although this could be limited by small sample size in the shorter PD duration groups (Table 3). In contrast, probable RBD without probable SW was consistently associated with higher odds of PD across disease duration groups (≤4 years: OR, 6.47; 95% CI, 4.33-9.60; 4.1-8 years: OR, 5.35; 95% CI, 3.33-8.57; >8 years: OR, 7.11; 95% CI, 4.43-11.41) (Table 3).

**Table 3.** Joint Analysis of Probable Sleep Behavior Disorders in Association With Parkinson Disease by Years Since Diagnosis^a^

Time since diagnosis, y	Probable sleep behavior disorders			
	Neither	Probable SW only	Probable RBD only	Probable SW and probable RBD
**>8 y**				
Case No./total No.%	42/22 763 (0.18)	2/107 (1.87)	33/2537 (1.30)	5/112 (4.46)
OR (95% CI)	1 [Reference]	12.05 (3.22-45.04)	7.11 (4.43-11.41)	12.95 (4.69-35.80)
**4.1-8 y**				
Case No./total No. %	50/22 771 (0.22)	1/106 (0.94)	27/2531 (1.07)	1/108 (0.93)
OR (95% CI)	1 [Reference]	6.27 (1.32-29.81)	5.35 (3.33-8.57)	3.90 (0.78-19.67)
**≤4 y**				
Case No./total No. %	53/22 774 (0.23)	0/105 (0)	41/2545 (1.61)	2/109 (1.83)
OR (95% CI)	1 [Reference]	NA	6.47 (4.33-9.60)	8.39 (2.46-28.61)

Abbreviations: NA, not applicable; OR, odds ratio; RBD, rapid eye movement sleep behavior disorder; SW, sleepwalking.

^a^ Model adjusted for age, smoking status (never, past, or current smoker), body mass index, calculated as weight in kilograms divided by height in meters squared (<21, 21-24.9, 25-29.9, 30-34.9, or ≥35), alcohol consumption (0, 1-9.9, 10-19.9, 20-29.9, or ≥30 g/d), caffeine consumption (mg/d), hypertension (yes or no), diabetes (yes or no), total sleep duration (<6 hours, 6-8 hours, >8 hours), excessive daytime sleepiness (rarely or ever, some days, or most days), hypnotics use (yes or no), antidepressant use (yes or no), and restless legs syndrome (yes or no). Missing values indicators for individuals missing information on a covariate were used.

## Discussion

To our knowledge, this is the first population study examining the association of SW with a major neurological disease with a general population as a reference group. We found that probable RBD with probable SW had a stronger association with PD than probable RBD alone.

SW occurs most often during arousal from the non-REM sleep phase. It manifests as a complex disorder, involving motor dyscontrol, mental confusion, and amnesia.^14^ Pathologic changes occasionally occur in the ventral mesopontine junction, which contains dopaminergic neurons^14^ and thus shares a common pathway with PD development. Furthermore, a genome-wide parametric linkage analysis for SW suggested a candidate region on chromosome 20, which includes the adenosine deaminase gene.^15^ Difference in adenosine metabolism could be associated with A_1_ and A_2A_ adenosine receptor antagonism, which has been shown to directly interact with dopaminergic signaling in animal studies.^16^

RBD is one of the strongest prodromal nonmotor symptoms for PD that has been identified to date.^3^ The RBD-PD association could be explained by α-synuclein deposition, a common pathology in both diseases.^3,17^ When assessed by questionnaire, participants may not be able to correctly separate SW from other nighttime movements caused by different etiologies, such as RBD.^18^ Questionnaire-assessed SW had a sensitivity of 100% and specificity of 86% relative to polysomnography confirmed cases.^10^ In a previous polysomnography study, 80% of patients with PD and a history of SW had co-occurring RBD.^7,8^ Consistently, 72.8% of patients in our cohort with PD and probable SW also had probable RBD. Analyses were done separately for single or both parasomnias in this study to address the potential misclassification associated with this low specificity.

Based on our results, a longer duration of PD was associated with probable SW and with a stronger association between probable SW and PD than between probable RBD and PD. This could be because of chance, given that there were fewer cases in the shorter PD duration groups. However, this observation is consistent with a previous study conducted in patients with PD, which found that individuals with longer disease duration were more likely to have SW.^5^ Arousal-related disorders, including SW, were more common among patients with PD who had longer treatment, greater severity of disease, and more depression and anxiety.^7,8^ Patients with PD who had more severe cognitive impairment had a higher risk of SW but did not have a higher risk of probable RBD.^8^ Progressive neurodegeneration and changes in neurotransmitter levels may further influence arousal regulation during the progression of PD. While RBD has been recognized as a prodromal symptom of PD, SW appears to be associated with symptomatic PD and associated sleep disruption.

Sleep disruption in PD is common. A previous survey reported that 64% of individuals with PD had sleep problems.^19^ Sleep disruption is multifactorial; is sometimes considered as a nonmotor symptom of PD; is sometimes attributed by patients to nighttime tremor, neuropsychiatric symptoms, or postural discomfort that is difficult to counteract because of bradykinesia; and has been hypothesized to also relate to circadian disruption. Even early in the disease, patients with PD may have an altered *BMAL1* expression and melatonin secretion.^19^ Patients with PD may have reduced amplitude of melatonin secretion, particularly those who reported sleepiness.^20^ Patients with RBD and PD have altered expression of several genes involved in circadian rhythm regulation including human period gene 1 (*PER1*)*, PER2*, and *PER3*, a trend for the later dim light onset of melatonin secretion.^21^ As dysregulation of the circadian control of sleep may lead to more fragmented sleep,^22^ sleep fragmentation of both REM and NREM sleep may be a major contributing factor to the higher frequency of probable SW and probable RBD in patients with PD. Although sleep fragmentation was not assessed in this study, we observed a higher percentage of participants with SW and RBD having excessive daytime sleepiness and either shortened (<6 hours) or prolonged (>8 hours) sleep duration.

### Limitations

Compared with previous studies on SW and PD, this study is the first to include non-PD control participants from a large cohort.^5^ However, it does have limitations. One major limitation of this study is the questionnaire-based method for assessing sleep disorders, which could introduce misclassification, as addressed previously in this article. However, the Mayo Sleep Questionnaire has been validated against clinical diagnosis and proved appropriate for use in large, population-based studies.^2,3,17,23^ Due to the cross-sectional nature of this study, we are unable to make conclusions about the temporal relationships between probable SW and PD.^10^ Evidence from future prospective studies is needed to address the value of SW as a potential prodromal marker for PD. Information on the childhood history of SW was not collected, limiting our ability to examine the potential early-life risk predisposition. Of note, the HPFS cohort consists of male health professionals who are predominantly White and who, at the time of this study, had a mean (SD) age of 76 (7.4) years, younger than the age with peaked prevalence globally (85-89 years).^1^ Thus, results from this study might not be able to generalizable to other populations with different characteristics.

## Conclusions

In this study, the presence of parasomnia, including both probable RBD and probable SW, was more common among men with PD, relative to those without PD. Prospective cohort studies in which long-term follow-up data are available with a reasonable number of SW and PD cases are warranted for better understanding the association.

## Notes

Supplement. **eFigure.** Flowchart of the Study Population **eTable 1. **Model Fitness Assessed by Bootstrap Validation **eTable 2. **Number of Missing Observations for Each Covariate Used in the Analyses **eTable 3. **Baseline Characteristics of the Included vs Excluded Population **eReferences.**
