# Supplementary material for: Association of Sleepwalking and REM Sleep Behavior Disorder With Parkinson Disease in Men
Source: JAMA Netw Open. 2021 Apr 13;4(4):e215713. doi: 10.1001/jamanetworkopen.2021.5713 (PMC8044732; doi:10.1001/jamanetworkopen.2021.5713)
Supplement: Supplement. — eFigure. Flowchart of the Study Population eTable 1. Model Fitness Assessed by Bootstrap Validation eTable 2. Number of Missing Observations for Each Covariate Used in the Analyses eTable 3. Baseline Characteristics of the Included vs Excluded Population eReferences. [file jamanetwopen-e215713-s001.pdf]

Supplemental Online Content

Zhang X, Molsberry SA, Pavlova M, Schwarzschild MA, Ascherio A, Gao X. Association of sleepwalking and REM sleep behavior disorder with Parkinson disease in men. *JAMA Netw Open*. 2021;4(4):e215713. doi:10.1001/jamanetworkopen.2021.5713

eFigure. Flowchart of the Study Population

eTable 1. Model Fitness Assessed by Bootstrap Validation

eTable 2. Number of Missing Observations for Each Covariate Used in the Analyses

eTable 3. Baseline Characteristics of the Included vs Excluded Population

eReferences.

This supplemental material has been provided by the authors to give readers additional information about their work.

© 2021 Zhang X et al. *JAMA Network Open*.

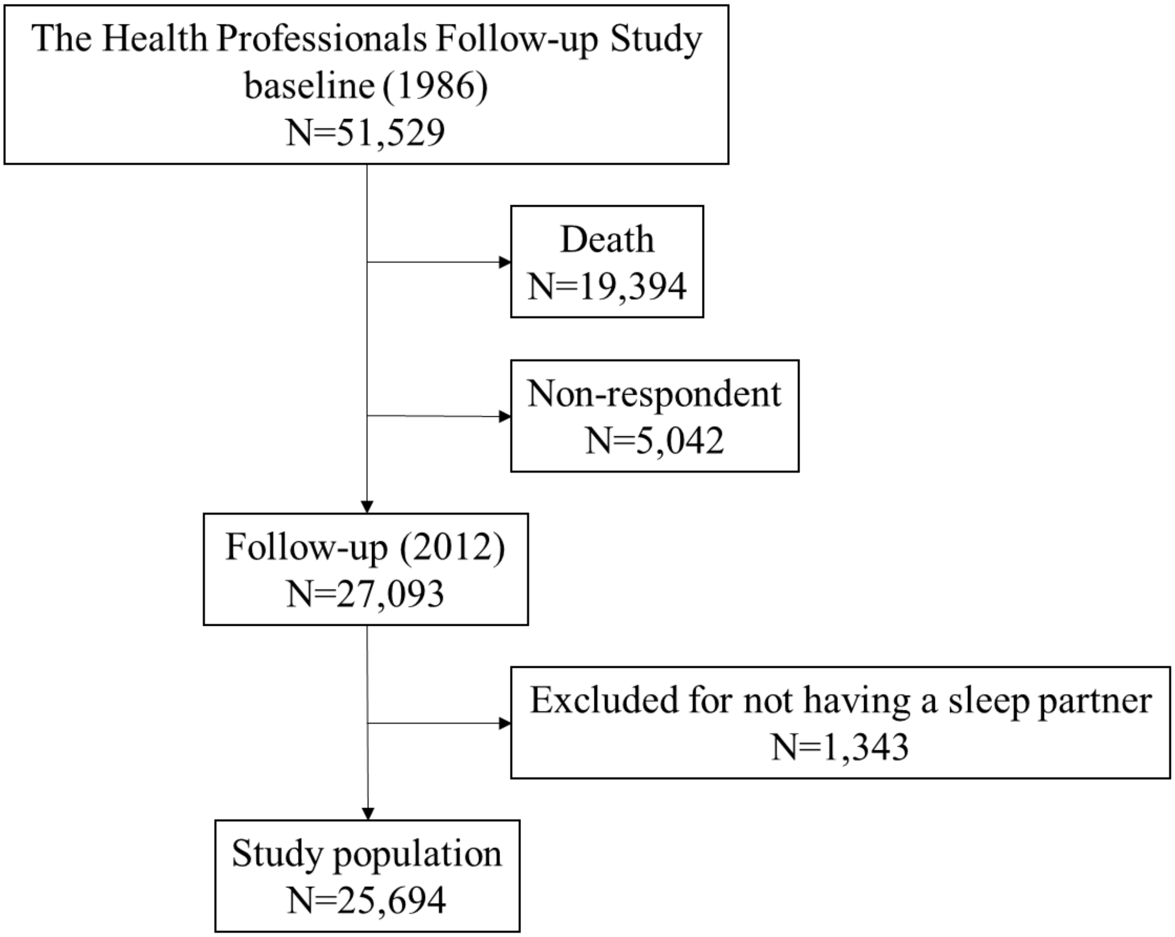

eFigure. Flowchart of the Study Population

© 2021 Zhang X et al. *JAMA Network Open*.

eTable 1. Model Fitness Assessed by Bootstrap Validation.

**Models in the manuscript**	**Optimism-corrected C-statistics**
All participants	0.794
Stratified by Parkinson disease duration	
>8 y	0.851
4.1-8 y	0.773
≤ 4y	0.739

Independent variables included probable sleep behavior disorders (pSW and pRBD), age, smoking status (never, past, or current smoker), body mass index (<21, 21–24.9, 25–29.9, 30–34.9, or ≥35 kg/m2), alcohol consumption (0, 1–9.9, 10–19.9, 20–29.9, or ≥30 g/d), caffeine consumption (mg/d), hypertension (yes/no), diabetes mellitus (yes/no), total sleep duration (h), excessive daytime sleepiness (rarely or ever, some days, or most days), hypnotics use (times/wk), antidepressant use (yes/no), and restless leg syndrome (yes/no).

© 2021 Zhang X et al. *JAMA Network Open.*

eTable 2. Number of Missing Observations for Each Covariate Used in the Analyses

**Variable**	**Missing no.**
Age	0
Body mass index	1399
Smoking status	1410
Alcohol consumption	4725
Caffeine consumption	4725
Hypertension	0
Diabetes mellitus	0
Total sleep duration	3932
Excessive daytime sleepiness	3947
Hypnotics use	3951
Antidepressant use	0
Restless leg syndrome	6450

© 2021 Zhang X et al. *JAMA Network Open.*

eTable 3 Baseline Characteristics of the Included vs Excluded Population.

	Included (n=25694)	Excluded (n=1343)
Age, y*	75.6 (7.4)	79.2 (8.2)
Body mass index, kg/m^2	24.6 (7.1)	25 (6.2)
Smoking status		
- Never, %	55.0	54.7
- Past, %	42.4	42.0
- Current, %	2.6	3.4
Alcohol consumption, g/d	13 (15.8)	12.6 (16.8)
Caffeine consumption, mg/d	120 (136)	129 (136)
Hypertension, %	49.4	51.2
Diabetes mellitus, %	11.6	12.6
Total sleep duration		
- Less than 6h, %	2.5	5.0
- 6-8h, %	86.1	78.8
- Over 8h, %	11.5	16.2
Excessive daytime sleepiness, %	11.0	13.5
Hypnotics use, %	14.8	18.4
Restless leg syndrome, %	4.4	5.0

Values are means (SD) for continuous variables; percentages for categorical variables, and are standardized to the age distribution of the study population.

* Value is not age-adjusted.

© 2021 Zhang X et al. *JAMA Network Open.*
